# The importance of mesorectum motion in determining PTV margins in rectal cancer patients treated with neoadjuvant radiotherapy

**DOI:** 10.1093/jrr/rrz092

**Published:** 2019-12-19

**Authors:** Zumre Arican Alickikus, Ahmet Kuru, Barbaros Aydin, Dogukan Akcay, Ilknur Bilkay Gorken

**Affiliations:** Department of Radiation Oncology, Dokuz Eylul University Faculty of Medicine, İzmir 35340, Turkey

**Keywords:** Rectal cancer, radiotherapy, mesorectum, dosimetric changes

## Abstract

New precision radiotherapy (RT) techniques reduce the uncertainties in localizing soft and moving tumors. However, there are still many uncontrollable internal organ movements. In our study, patients who underwent neoadjuvant chemoradiotherapy (NA-CRT) for rectal cancer were evaluated to determine inter-fraction mesorectum motion and dosimetric changes. Fourteen patients treated with NA-CRT for rectal cancer between 2014 and 2016 were included in the analysis. The mesorectum and clinical target volume (CTV) were delineated on planning computed tomography (CT) and cone-beam CT (CB-CT) scans. After planning with a volumetric modulated arc therapy (VMAT) plan, re-planning was performed on all CB-CTs. Finally, the volumetric and dosimetric changes of PTV and mesorectum were evaluated in all CB-CTs compared with the initial CT and VMAT plans. The geometrical center of mesorectum volume in CB-CTs had moved 1 (0.2–6.6), 1.6 (0.2–3.8) and 1.6 (0–4.9) mm in the *x*, *y* and *z*-axis respectively compared with the initial CT. The dosimetric parameters of PTV including D2, D95 and D98 on CB-CT showed a median 47.19 (46.70–47.80), 45.05 (44.18–45.68) and 44.69 (43.83–45.48) Gy and median 1% (1–2), 0% (0–2) and 1% (0–2) dosimetric change compared with the initial VMAT plan. In our study, we have shown that the mesorectum has moved up to 20 mm in the lateral and anterior–posterior direction and almost 10 mm in the superior/inferior direction during RT, causing a median of ~2% change in dosimetric parameters. Therefore, these movements must be considered in determining PTV margins to avoid dosimetric changes.

## INTRODUCTION

Colorectal cancers constitute 8% of all cancers and are one of the leading causes of cancer-related deaths in the world [1]. Post-treatment local recurrence is the most prominent cause of treatment failure in rectal cancers accounting for nearly one-third of colorectal cancers [2,3]. Therefore, the parameters that could affect treatment success in local control and local treatments are especially important in rectal cancer [4].

The main treatment modality for rectal cancer is surgical resection, and most of the patients require extensive surgical procedures such as abdominoperineal resection (APR) or anterior resection (AR). The mesorectum and outermost perirectal fascia act as a barrier to the spread of cancer and constitute the surgical ‘circumferential margin’. In surgical procedures for rectal cancer, the importance of the mesorectum results from the fact that lymph node or occult metastases can occur at its level, which may not be eliminated in case of incomplete or partial resection and should undergo total excision [5,6]. Failure to remove the mesorectal fat entirely may explain part of observed local and distant recurrences. Total mesorectal excision (TME), introduced by Heald and Ryall in 1986, is now the standard surgical approach in the treatment of rectal cancer [2]. The recurrence rates before the use of TME were around 15–30% and decreased by up to 10% after TME. These outcomes suggest the importance of treating the entire mesorectum, and neoadjuvant chemoradiotherapy (NA-CRT) has become a treatment approach with increasing importance in the management of rectal cancer treatment [2]. Randomized controlled studies support the important role of NA-CRT in the presence of a T3–T4 and distal tumors that require APR. In T1–T2 tumors with node-positive disease there is a risk of failure to achieve negative circumferential resection margin [7]. Although neoadjuvant radiotherapy (RT) and adjuvant RT do not exhibit any differences in survival according to long-term outcomes, neoadjuvant RT was found to be superior in terms of local control rates [8, 9]. According to a German Phase III Study Group, the local recurrence (7.1 vs 10.1%, *P* = 0.048) and sphincter preservation rates (39 vs 19%, *P* ≤ 0.05) were significantly higher in the neoadjuvant RT arm compared to adjuvant RT [9]. Moreover, the use of radiosensitizers and concomitant chemotherapy that would be added to increase systemic control was shown to contribute to the increase in local control and disease-free survival in neoadjuvant RT patients [10]. In a randomized Polish study that compares conventional fractionated CRT (50.4 Gy/28 fractions with concomitant 5-fluorouracil (5-FU) and folinic asid) and short fractionated RT (25 Gy/5 fractions), the rate of pathological complete response (pCR) was 15% in the CRT arm and 1% in the short-fractionated RT arm [11]. As a result, in practice, neoadjuvant RT with or without concomitant chemotherapy followed by surgical TME should be the standard approach in locally advanced rectal cancer since it increases the response rate and local control in addition to enabling sphincter preservation [8, 9, 12, 13].

In the management of rectal cancer treatment with neoadjuvant RT, the first step is to determine the clinical target volume (CTV_primary_) on computed tomography (CT) scan slices that encompass the entire mesorectum and the primary tumor and the nodal CTV for the related regional lymph nodes (presacral, external and internal iliac, obturator, inguinal) based on the clinical examination, endoscopic and radiologic examination [CT/PET-CT (positron emission CT), MRI (magnetic resonance imaging)]. These target volumes are determined and contoured according to the localization and clinical stage of the disease. This is followed by the determination of the planning tumor volume (PTV) by applying a safety margin of 1–1.5 cm to the CTV due to the physiological movement of adjacent organs and set-up errors. Today, it is possible to reduce the dose for surrounding normal tissues while administering the highest dose to the target tumor volume with sharp dose gradients due to advanced RT techniques such as intensity-modulated radiation therapy (IMRT) and volumetric modulated arc therapy (VMAT). Mesorectum is of foremost importance as it is the target volume in NA-CRT. Movement of the rectum and mesorectum can cause dose uncertainties [14]. Moreover, organ movements may cause changes in planned doses of patients becuase of sharp dose gradients. The risk of a serious side-effect can be seen due to delivering doses higher than planned to the surrounding normal tissues, and a loss of local control might be encountered due to delivering doses lower than planned to the target volume as a result of mesorectum movement. It is important to generate solutions to increase treatment accuracy by minimizing uncertainties that could stem from organ movements with the use of image-guided radiation therapy (IGRT) [15].

The aim of this study was to investigate the variations in mesorectum movements and doses delivered to the target volume, using cone-beam CT (CB-CT) images obtained throughout the treatment period from the locally advanced rectal cancer patients who underwent NA-CRT.

## MATERIALS AND METHODS 

### Patient characteristics and CT simulation

The treatment data of 14 consecutive patients who had locally advanced tumors localized in the mid-rectum and received NA-CRT between March 2014 and February 2016 were evaluated in this study. Mid-rectum cancer was considered a tumor between 7 and 11 cm away from the anal verge on MRI scan. In our daily practice, tumor and lymph status are staged according to European Society of Gastrointestinal and Abdominal Radiology (ESGAR) criteria for radiological staging and response evaluation in locally advanced rectal cancer patients [16]. Therefore, in this study patients were staged according to radological Tumor, Node, Metastasis (TNM) staging system. Before the day of the simulation, hydration is ensured by providing fluids for the patients, at least 2 L/day. Then the patients undergo planning CT simulation following bowel emptying with the use of laxatives/purgatives for suitable bowel preparation. On the day of the simulation, all patients are provided 500 mL of oral hydration after emptying the bladder and asked to wait for 45 min to ensure optimal bladder fullness. The CT images from the first lumbar vertebra to one-third of the proximal femur are obtained in the supine position with arms up using suitable immobilization tools at a 3 mm slice interval and transferred to the treatment planning system. For all patients included in this study, CT simulation images were obtained with the ‘Siemens’ brand ‘Somatom’ model computerized tomography (CT) device and transferred to the Eclipse v11 planning system.

Daily CB-CT images obtained during treatment were used for re-planning and dose re-calculation. Informed consent was obtained from all individual participants included in the study. Ethical approval for this study was obtained from the Non-interventional Studies Ethics Committee of Dokuz Eylul University Medical Faculty (Ethics Committee approval number: 2018/29-26).

### Target volume and normal tissue contouring

All target tumor volumes (Gross tumor volume (GTV), CTV) and normal tissues (bladder, sigmoid, rectum and small bowel) were contoured by a radiation oncologist on the slices obtained in CT simulation. In our clinical practice, total CTV encompasses the CTV mesorectum defined as whole mesorectum in CT/MRI images which included GTV primary (if the tumor extends the mesorectal fascia CTV mesorectum contours are expanded ~ 1 cm beyond the primary tumor) and CTV lymphatics which included external–internal iliac and presacral lymph nodes with 0.7 cm margins as suggested in guidelines [17,18]. We believe that anteriorly located advanced T3 tumors, especially with any detected pelvic node with short-axis diameter 5–8 mm and one of the suspicious criteria as described in the literature (irregular border/heterogeneous signal in MRI/round shape), may have the risk for subclinical disease [16]. Therefore, external iliac lymph nodes were included in CTV lymphatics in patients with T4 tumors and anteriorly located advanced T3 tumors including suspicious criteria for pelvic lymph nodes. One patient with T2 tumor had a radiologically proven positive pelvic lymph node. In this case, external iliac lymphatics were also included in CTV lymphatics.

PTV was created by adding a 10 mm safety margin to CTV in all directions for internal organ motion and setup errors.

### Treatment planning and delivery

External beam radiation therapy (EBRT) was delivered in 25 fractions of 180 cGy per day, for a total dose of 4500 cGy with the VMAT technique. The treatment was administered using the TrueBeam STx Linac device. In addition to the kilovoltage (kV) imaging performed each day, CB-CT images were obtained each day for the first 3 days and then at least once a week throughout the treatment period followed by IGRT. Patients who had at least 4 CB-CT imaging during RT treatment were included in this study. Optimization criteria for PTV and organs at risk were as follows: (D_98_) > 98%, (D_2_) <105% for PTV and bladder mean dose <35 Gy and volume of the small bowel receiving a dose of 45 Gy (V_45_) <195 cc.

During the treatment course, all patients are trained to drink at least 2 L/day of water and eat an appropriate diet for bowel and bladder consistency. On each day of treatment, the patient was asked to drink 500 mL of water after emptying the bladder and wait for 45 min to ensure optimal bladder fullness.

### Mesorectum contouring

The entire mesorectum was separately contoured on the planning CT (P-CT) without changing the previously determined target volumes and treatment plans. On CB-CT the rectum was contoured cranially until the sigmoid flexure location. The upper rectum is intraperitoneally surrounded by peritoneum except for a small segment posteriorly and the middle rectum is covered by peritoneum only anteriorly. The mesorectum was considered as an adipose tissue surrounding the rectum and was contoured within the limits of the mesorectal fascia, from the sacral promontorium to the level at which the levator ani enters the rectal wall. In case of uncertainty, bifurcation of the internal mesenteric artery in the sigmoid artery and the superior rectal artery was considered in re-contouring the cranial limit of mesorectum. The anterior limit of the mesorectum was determined as the mesorectal fascia if visible or posterior border of the anterior pelvic organs. The mesorectum was contoured within the limits of the mesorectal fascia, from the sacral promontorium to the level at which the levator ani enters the rectal wall. The mesorectum was always contoured by a single radiation oncologist and checked by a radiation oncology specialist for consistency purposes.

### Matching and re-contouring

In order to determine the mesorectum movements in CB-CT images obtained during RT treatment of the patients, CB-CT images were matched with P-CT images on the Eclipse v11 planning system. After the matching process, mesorectum contours on P-CT images were transferred to each CB-CT image and the entire mesorectum was re-contoured on CB-CT images.

### Re-planning and dose re-calculation

CB-CT images were used for re-planning, and the patients were planned to receive a total dose of 45 Gy in 25 fractions using VMAT. VMAT optimization was performed with 2 full arcs with 10 MV photon energy. Dose calculations were performed using the analytical anisotropic algorithm (AAA). CB-CT images were selected from the off-line review segment of the Aria 11 system to evaluate the mesorectum shifts, volume variations and effects of the dose on the mesorectum according to P-CT. D_98_, D_95_ and D_2_ doses for the mesorectum were recorded after the dose calculations according to P-CT and each CB-CT. The geometric center of the mesorectum according to P-CT and each CB-CT obtained after matching was calculated according to the Cartesian coordinate system (Δ*x*, Δ*y*, Δ*z*). The shift in the geometric center of the mesorectum was calculated from each CB-CT and the displacement of this center according to P-CT (mm) and the shift direction of the mesorectum [right–left (ΔR–ΔL), anterior–posterior (ΔA–ΔP) and inferior–superior (ΔI–ΔS)] were determined. Finally, the median mesorectal volume was calculated from each CB-CT and the percentage change according to P-CT was determined.

### Statistical analysis

Statistical analysis of the data was performed using the Statistical Package for the Social Sciences (SPSS) version 23. Nonparametric tests (Chi-square, Mann-Whitney U) were used for intergroup comparisons and the Spearman Rank Correlation test was used for correlation analysis. The level of significance was accepted as *P* < 0.05.

## RESULTS 

### Patient and tumor characteristics

The median age of the 14 patients in the study was 64.5 (43–85) years. Only the patients who had a tumor localized in the mid-rectum were included in the study to have consistent target volumes. Ninety-three percent of the patients had radiological/clinical stage T3 tumors, whereas 86% of the patients had metastasis to lymph nodes. Patient and tumor characteristics are shown in Table 1.

**Table 1:** Patient and tumor characteristics (*n* = 14)

**Characteristics**	***n***	**%**
**Gender**		
Male	11	78
Female	3	22
**T Stage**		
T2	1	7.1
T3a	-	-
T3b	9	64.3
T3c	3	21.5
T3d	1	7.1
**N Stage**		
N0	2	14.2
N+	12	85.8

### Interfractional motion of the mesorectum and geometric center shift

**Fig. 1:**
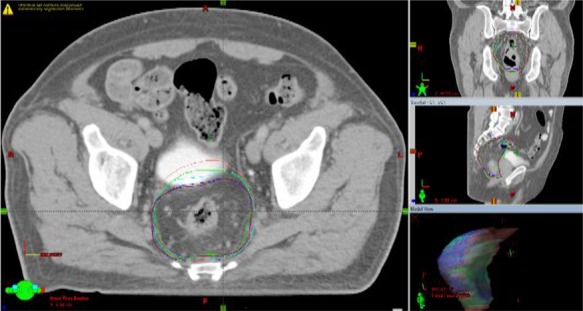
Changes in mesorectum contours on cone-beam CT images matched to planning CT scans (P-CT, CB-CT 1, CB-CT 2, CB-CT 3, and CB-CT 4)

Seventy planning and CB-CT images were used to calculate the change in mesorectum contours in each direction and the shift in geometric centers. In CB-CT, the median right and left lateral movement of the mesorectum was 4.9 (1.3–19.8) and 5.0 (0.2–12.5) mm, the median anterior and posterior movement of the mesorectum was 12.4 (4-26.8) and 4.65 (1.5-9.5) mm, and the median superior and inferior movement of the mesorectum was 0.8 (0–6) and 1.9 (0–9 mm) respectively according to initial planning-CT (Figure 1).

The median movement of the geometric center of mesorectal volume in the *x*, *y* and *z* axes was 1 (0.2–6.6), 1.6 (0.2–3.8) and 1.6 (0–4.9) mm respectively in CB-CT images in reference to the p-CT scans (Table 2).

**Table 2:** The median shift in the geometric center of the mesorectal volume of patients in *x*, *y* and *z* axes

Patient number	T stage	∆*x* (mm)	∆*y* (mm)	∆*z* (mm)
1	T2	0.3	0.2	0.2
2	T3b	6.6	2.4	0.5
3	T3b	1.4	3.1	0.4
4	T3b	1.5	1.8	3.6
5	T3b	0.3	0.9	0.0
6	T3b	0.7	1.9	2.2
7	T3b	1.6	2.4	0.7
8	T3b	2.8	1.6	4.9
9	T3b	0.2	0.7	2.4
10	T3b	2.5	3.8	3.3
11	T3c	1.0	1.3	1.9
12	T3c	0.2	1.6	0.5
13	T3c	1.0	0.7	2.8
14	T3d	0.8	1.0	1.2
Median		1.0	1.6	1.6

**Table 3:** Rectal cancer IGRT studies showing the magnitude of motion and region studied

**Reference**	**No of patients**	**Imaging method**	**Motion: magnitude and direction (mm)**	**Region studied**
Brierley *et al*. [19]	17	p-CT scans at weeks 1, 3 and 5	Left-right (mean): 1.2; anterior–posterior (mean): 0.7; superior–inferior (mean): 4.2	Rectum and mesorectum
Tournel *et al*. [20]	10	CT scan (tomotherapy)	Left-right (mean): 1.6; anterior–posterior (mean): 2.0; superior–inferior (mean): 3.2	Mesorectum
DeNittis *et al*. [23]	9	Weekly CT scans	Left–right (mean): 4.4; anterior–posterior (mean): 6.0	Rectum
Lee *et al*. [24]	9	Daily cone-beam CT	Lateral (max): 26; anterior–posterior (max): 37.7	Rectum
Ippolito *et al*. [25]	20	Weekly CT scans	Maximum deviation (mean): 74 cc	Rectum
Nuyttens *et al*. [26]	10	Weekly CT scans	Cranial (mean): 10; caudal (mean): 15	Rectum, perirectal tissues and regional lymphatics

**Table 4:** Mesorectum volumes and the mean % change in the mesorectal volume in cone beam-CT considering the planning-CT [(+): increase, (−): decrease]

**Patient number**	**T stage (AJCC 7th edition)**	**Mesorectum volume (cc)**					**Change in volume (%)**
		**Planning-CT**	**CB-CT-1**	**CB-CT-2**	**CB-CT-3**	**CB-CT-4**	
1	T2	389.50	417.90	368.60	372.30	364.30	−2.24
2	T3b	337.00	379.00	353.60	368.10	386.40	+10.32
3	T3b	375.50	349.60	373.80	357.10	349.10	−4.82
4	T3b	373.40	423.50	410.40	395.50	443.10	+11.98
5	T3b	379.30	407.20	344.50	397.20	344.60	−1.56
6	T3b	339.20	339.80	369.80	379.20	326.00	+4.27
7	T3b	265.90	289.10	291.90	298.60	277.50	+8.79
8	T3b	372.00	377.30	368.60	367.70	351.60	−1.53
9	T3b	276.90	312.70	262.00	280.00	325.70	+6.57
10	T3	272.40	385.10	353.10	334.20	314.10	+27.25
11	T3c	218.40	273.60	298.20	307.30	297.30	+34.66
12	T3c	351.70	401.00	437.90	465.10	398.10	+20.99
13	T3c	330.50	340.20	398.90	342.20	339.00	+7.44
14	T3d	374.00	400.30	415.40	372.80	369.10	+4.12
Median		345.45 (218-389)	378.15 (273–424)	368.60 (262–438)	367.90 (280–465)	346.85 (277–443)	+7.01
Mean		332.55	364.02	360.48	359.81	348.99	+5.76

Reviewing the median shift in the geometric center of the mesorectal volume in the *x*, *y* and *z* axes according to T stage in Table 2, it is seen that the median shift of the geometric center of the mesorectal volume in the *x*, *y* and *z* axes in a patient at clinical stage T2 was 0.3, 0.2 and 0.2 mm respectively. On the other hand, the median shift in the geometric center of the mesorectal volume in the *x*, *y* and *z* axes in 9 patients at clinical stage T3b was 1.5 (0.2–6.6), 1.9 (0.7–3.8) and 2.2 (0–4.9) mm respectively. Moreover, the median shift in the geometric center of the mesorectal volume in the *x*, *y* and *z* axes in 4 patients at clinical stage T3c-T3d was 0.9 (0.2–1.0), 1.15 (0.7–1.6) and 1.55 (0.5–2.8) mm respectively. Rectal cancer IGRT studies showing the magnitude of motion and region are shown in Table 3.

**Fig. 2:**
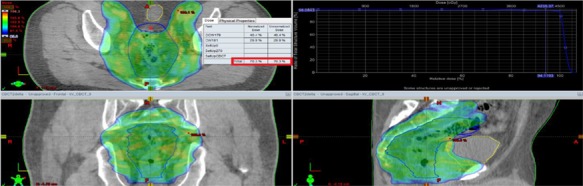
Re-planning with VMAT on cone-beam CT and dose distribution for the mesorectal volume. Mesorectum contour has moved beyond the planned 95% isodose line on CB-CT with an up to 30% dose decrease according to planned dose.

**Table 5:** The percentage (%) of changes in the mesorectum mean doses according to VMAT planning calculated using cone beam-CT images [(+): increase. (−): decrease). p-D*x*: *x* doses in planning CT. CB-CT-D*x*: *x* doses in cone beam-CT.

	**Mesorectum doses (Gy)**									
**Patient number**	**T stage**	**p-D98**	**CB-CT-D98**	**Change in dose (%)**	**p-D95**	**CB-CT-D95**	**Change in dose (%)**	**p-D2**	**CB-CT-D2**	**Change in dose (%)**
1	T2	45.0	45.3	−1	45.2	45.6	−1	46.7	47.5	−2
2	T3b	44.8	44.2	+1	45.0	44.9	+1	46.6	47.0	−1
3	T3b	44.9	44.3	+1	45.0	45.4	−1	46.7	47.8	−2
4	T3b	44.8	43.8	+2	45.0	44.2	+2	46.8	46.7	0
5	T3b	44.9	44.6	+1	45.0	44.9	0	46.8	47.1	−1
6	T3b	44.9	44.2	+2	45.0	44.5	+1	46.9	47.1	0
7	T3b	45.0	44.2	+2	45.1	44.5	+1	46.7	46.7	0
8	T3b	45.0	45.5	−1	45.1	45.7	−1	46.7	47.4	−1
9	T3b	45.0	44.8	+1	45.1	45.2	0	46.7	47.3	−1
10	T3b	45.0	45.1	0	45.1	45.3	0	46.8	47.3	−1
11	T3c	45.1	45.1	0	45.2	45.3	0	47.0	47.3	−1
12	T3c	44.8	45.1	−1	44.9	45.2	−1	46.8	47.2	−1
13	T3c	45.0	44.9	0	45.1	45.2	0	47.0	47.2	+1
14	T3d	45.0	44.8	0	45.1	45.1	0	46.8	47.1	−1
Median		45.00 (44.8–45.1)	44.8 (43.8–44.8)	1 (0–2)	45.1 (44.9–45.2)	45.2 (44.2–45.1)	0 (0-2)	46.8 (46.6–47.0)	47.2 (46.7–47.1)	1 (0–2)

### Interfractional mesorectal volume variation

The mesorectal volume exhibited a median change of 7.01 (4.8–34.6) cc in CB-CT scans as compared to p-CT scans, where there was 7% (4.1–34.6) increase in 10 patients and 1.5% (1.5–4.8) decrease in 4 patients (Table 4).

### Interfractional mesorectal dose variations

According to the re-planning calculated by using CB-CT images, the median D_2_, D_95_ and D_98_ values for the mesorectum were 47.19 (46.70–47.80), 45.05 (44.18–45.68) and 44.69 (43.83–45.48) Gy respectively (Table 5).

Moreover, a few sections of the mesorectum volume was seen beyond the planned 95% isodose line on CB-CT and meaningful dose decrease was determined according to planned dose as shown in Fig. 2. The median dose change of 1 (1–2), 0 (0–2) and 1% (0–2) was observed in D_2_, D_95_ and D_98_ values for the mesorectum according to CB-CT as compared to p-CT.

### Interfractional bladder volume variation

The median bladder volume was 234 (80–350) cc in P-CT. The mean and median bladder volume changes in CB-CT scans compared to P-CT are shown in Table 6.

**Table 6:** Bladder volume changes in CB-CT according to P-CT

	**Median volume (cc)**	**Median volume change (%)**	**Mean volume change (%)**
**CB-CT 1**	196 (67–328)	−13 (Range −44 to +113)	−2
**CB-CT 2**	162 (48–290)	−37 (Range −61 to +59)	−22
**CB-CT 3**	99 (47–320)	−34 (Range −75 to +15)	−32
**CB-CT 4**	165 (43–410)	−30 (Range −68 to +143)	−4
**Total change (%)**	-	−19 (Range −52 to +42)	−15

## DISCUSSION 

It is important to define an adequate and suitable PTV that encompasses the CTV, the primary tumor volume, rectum, regional lymphatics and the mesorectum in neoadjuvant RT for rectal cancer. The mesorectum is the most important and largest part of the CTV, and it exhibits internal motion independent of the bony structures due to its localization and the physiological movement of neighboring organs such as the rectum and bladder. Furthermore, it is visible in both megavoltage (MV) imaging and kilovoltage (kV) imaging. Brierley *et al*. conducted a study on 17 patients who received neoadjuvant RT due to stage II–III rectal cancer and evaluated rectal motion [19]. During RT, the greatest movement of the mesorectum occurred in the anterior and posterior directions and in the craniocaudal direction for GTV. They found a mesorectum motion of 8 mm right–left, 8 mm anterior and 9 mm in the posterior direction. Similarly, our study also showed that the greatest movement was in the anterior and posterior direction, and the median right and left lateral movement of the mesorectum was 4.9 (1.3–19.8) and 5.0 (0.2–12.5) mm, the median anterior and posterior movement of the mesorectum was 12.4 (4–26.8) and 4.65 (1.5–9.5) mm, and the median superior and inferior movement of the mesorectum was 0.8 (0–6) and 1.9 (0–9) mm respectively in CB-CT in reference to the p-CT. In a study by Tournel *et al*. conducted in 2008, they investigated intrafractional internal organ movement using daily MV-CT images of 10 rectal cancer patients [20]. They found that the mesorectum movements were 0.1 and 1.6 mm right and left, 2 and 0.4 mm anterior and posterior, 3.2 and 3.2 mm cranial and caudal. Consequently, it was emphasized that set-up margins could be minimized by determining intrafractional internal organ movements by conducting daily MV-CT scanning in rectal cancer patients who received RT with helical tomotherapy. In a systematic review carried out by Gwynne and Webster, inter- and intra-fractional movements of the rectum, bladder and mesorectum were reviewed [21]. They underlined that there could be a significant movement especially in the superior mesorectum, and this could be overcome by rectal filling. They also emphasized that the margin between CTV and PTV should be between 1 and 3.5 cm based on this movement. Furthermore, they suggested administering IGRT with CB-CT imaging weekly to determine internal organ motion. In our study, we demonstrated that the mesorectum moved 20 mm laterally and in the anterior/posterior direction and up to 10 mm in the inferior/superior direction throughout the treatment period, which led to median dose changes of up to 2% in D2, D95 and D98 values. Consequently, the actual dose delivered to the mesorectum was decreased and this situation may have an impact on clinical outcomes. In addition, the importance of sufficient PTV margins and IGRT was underscored once again. In a recent study, Yamashita *et al*. analysed organ movements by contouring the rectum and bladder from weekly CB-CT scans obtained during the planning and treatment period from 32 rectal cancer patients who received preoperative CRT [14]. The mean values (± SD) of the percentages of the rectum exceeding target volumes on CB-CTs of the patients in which the margins of 0, 3, 5, 7, 10 and 15 mm were added to the rectal CTV on planning CT were 20.7 ± 12.5%, 7.2 ± 8.3%, 3.9 ± 5.9%, 2.1 ± 3.9%, 0.7 ± 1.8% and 0.1 ± 0.3% respectively. In this study evaluating the movements that could stem from tumor shrinkage in addition to rectal fullness, it was consequently underlined that adding 10–15 mm safety margins to the rectal volume could be enough to avoid dose changes due to motion. In our study, the entire mesorectum that is at a considerable risk of submicroscopic disease and rich in blood vessels/lymph nodes including the rectal wall and its surrounding area was contoured to evaluate the movement–dose relationship in addition to the rectal volume variation due to tumor shrinkage. The median movement of the geometric center of mesorectal volume in *x*, *y* and *z* axes was 1 (0.2–6.6), 1.6 (0.2–3.8) and 1.6 (0–4.9) mm respectively in CB-CT images concerning the p-CT scans. Similar to the results of the study by Yamashita *et al*., the shift of the centroid was limited. Moreover, the median change in the total mesorectal volume was 7 (4.8–34.6) cc, including an increase of up to 7% and a decrease of 1.5% [14]. All these changes resulted in dose variations of up to 2% in D_95–98_ values for the mesorectum, which is important in terms of local recurrence and in D_2_ values that are important for normal tissues.

Nijkamp *et al*. conducted a study on 27 rectal cancer patients receiving hypofractionated RT in 2009 and investigated the intrafractional CTV and mesorectal movements using daily pretreatment CB-CT scans in addition to the effects of female/male gender on this motion because of the differences in the anatomy of the pelvis [22]. The researchers found that the greatest movement of the mesorectal volume was seen in the superior and anterior direction (~1–7 mm), and the displacement in females was 3 mm higher than that in males. The effect of gender on mesorectal movement was not investigated in our study, and despite 78% of patients being male, who could be predicted to have less mesorectal movement due to narrower pelvic structure as compared to females, the median mesorectal movement in the entire patient series was found to be 4.9–5.0 mm to the right and left, 12.4—4.6 mm in the anterior and posterior direction, and 0.8 and 1.9 mm in the superior and inferior direction, similar to the results of the study by Nijkamp *et al*. [22].

In the study by Brierley *et al*. of rectal cancer patients receiving neoadjuvant RT, they measured a median mesorectal movement in the anterior and posterior direction, and the primary tumor exhibited a mean displacement of 1.1 mm in the left and right direction, 0.9 mm in the anterior and posterior direction and 1.6 mm in the superior and inferior direction throughout the RT treatment period [19]. They did not evaluate the effect of the primary tumor stage on movement. We also did not evaluate the association between tumor stage and movement. However, it was observed that one patient with a clinical stage T2 tumor had the smallest displacement of the mesorectal contour, mesorectal volume and the geometric center of the target volume in all directions according to CB-CTs in comparison to p-CT. It was also found that 4 patients with T3c-T3d tumors had a smaller displacement of the mesorectal volume, geometric center and contour in all directions especially in the right–left lateral, superior–inferior and anterior directions in comparison to the patients with T3b tumors according to CB-CTs in comparison to p-CT. Accordingly, it can be inferred that internal organ movement can be more limited in patients with an intact mesorectum. However, further studies with a higher number of patients and with a homogenous T stage distribution are required to make a clear interpretation and more accurate inference.

## CONCLUSION 

In conclusion, the mesorectum is a structure that exhibits internal motion due to its localization and the physiological movement of neighboring organs such as the rectum and bladder. Therefore, in our study, we demonstrated that the mesorectum moved 20 mm laterally and in the anterior/posterior direction, and up to 10 mm in the inferior/superior direction, which led to median dose changes of up to 2% in D2, D95 and D98 values. The data we collected was consistent with the results of the other studies in the literature. The most interesting result was that the irradiated area could even exceed PTV margins due to interfractional variations in the mesorectal volume and motion direction. This could violate the main principle of RT, i.e. maximum dose in the tumor and minimum dose in normal tissue. Increased side-effects can be seen due to delivering doses higher than planned to the normal tissues, and a loss of local control can be encountered due to delivering doses lower than planned to the clinical target volumes as a result of volume variations due to mesorectum movement during RT. As a result of our study, we maintain that each clinic should specify their own PTV margins using reproducible simulation/treatment protocols by considering the movements of the mesorectum.
